# Heartworm (*Dirofilaria immitis*) infection in a leopard (*Panthera pardus pardus*) housed in a zoological park in north-eastern Italy

**DOI:** 10.1186/1756-3305-3-25

**Published:** 2010-04-08

**Authors:** Sandro Mazzariol, Rudi Cassini, Laura Voltan, Luca Aresu, Antonio Frangipane di Regalbono

**Affiliations:** 1 Dipartimento di Sanità Pubblica, Patologia Comparata e Igiene Veterinaria, Università degli Studi di Padova; Viale dell'Università, 16 - 35020, Legnaro (Padova), Italy; 2 Dipartimento di Scienze Sperimentali Veterinarie, Università degli Studi di Padova; Viale dell'Università, 16 - 35020, Legnaro (Padova), Italy; 3 Zoological Park Valcorba - 35020, Pozzonovo (Padova), Italy

## Abstract

Canine heartworm (cHW) disease is now recognised as potential cause of serious disease in cats and other felids, especially in endemic areas. In March 2009, a 23-years-old male African leopard (*Panthera pardus pardus*) housed in a zoological park located in the Province of Padova (Veneto Region), a cHW endemic area of the north-eastern Italy, died and was immediately necropsied. A cloth completely occluding the pyloric lumen was considered the presumptive cause of death. During necropsy, six nematodes (4 males and 2 females) were found within the right ventricle of the heart and the pulmonary artery. Diagnosis of HW (*Dirofilaria immitis*) infection was carried out by morphological features of adult worms and microfilariae, and then confirmed by detection of circulating HW antigens using a commercial SNAP kit (IDEXX Laboratories inc., USA). *D. immitis *infection was also confirmed by PCR amplification of the 5S ribosomal spacer region, performed on worm fragments and microfilaraemic blood samples obtained from the right ventricle of the heart. A glomerulonephritis of immuno-mediated origin and most likely associated with the HW infection is also reported. HW chemoprophylaxis and annual serological testing on wild felids housed outdoors in endemic cHW disease areas are recommended. This is the first diagnosis of *D. immitis *infection in an exotic felid in Italy.

## Findings

The African leopard (*Panthera pardus pardus*) occurs across most of the sub-Saharan region and as a tiny relict population in north Africa. As well as other *P. pardus *subspecies, it is currently listed as a critically endangered cat species in the IUCN red list of Threatened Animals. *Dirofilaria immitis*, the causative agent of canine heartworm (cHW) disease, is widespread in tropical, subtropical and some temperate regions. Transmission of this nematode occurs overall in the United States [1] and mainly in the southern European countries. In Italy, besides the hyperendemic areas along the Po River Valley, cHW infection chiefly concern the northern and central regions [2], while new autochthonous foci have recently been reported in southern Italy [3]. Although dogs are the natural hosts, cHW disease is now recognised as potential cause of serious disease in felids, especially in endemic areas. Both in domestic and non-domestic cat species, cHW disease is usually characterised by a low burden, while microfilaraemia is often absent otherwise transitory and with low intensity [4-6]. Although *D. immitis *infections have previously been detected in different species of wild or captive felids, e.g. in the wild cat (*Felis bangsi costariensis*) [5], tiger (*Panthera tigris*) [6,7], Clouded leopard (*Neofelis nebulosa*) [8,9], Golden cat (*Felis temminckii*) [10], bobcat (*Linx rufus*) [11], Black-footed Cat (*Felis nigripes*) [12], Snow leopard (*Uncia uncia*) [13], ocelot (*Leopardus pardalis*) [14], and African lion (*Panthera leo*) [15], to date the only report of cHW disease in *P. pardus *concern a single adult female specimen of *D. immitis *found in the heart of a wild-caught Black Panther in west Malaysia [16]. This report describes the first diagnosis of mature heartworms (*D. immitis*)infection in an exotic felid in Italy.

At the end of March 2009, a 23-year-old, 24 kg in weight, intact male African leopard housed in a zoological park of the Veneto Region (Province of Padova; 45°8' N, 11°50' E), died after a week of dysorexia and inappetence. The animal was immediately submitted for post-mortem examination at the Faculty of Veterinary Medicine of Padova. A cloth completely occluding the pyloric lumen and the proximal duodenum was considered the presumptive cause of death. Gastric dilation and a massive, locally extensive chronic gastritis with fibrosis and multifocal hemorrhagic ulcers along the big curvature in the fundic and pyloric region were found, due to the foreign body used by zoo keepers as an environmental enrichment. Other pathological findings were hepatic atrophy with multifocal necrosis, multifocal *ab ingestis *pneumonia, some follicular adenomas in both thyroid glands and fibrous osteodistrophic changes of the bones of the carpal joints, with osteophytes and multiple fractures of the lateral bones. Renal and other tissue samples for histopathology were collected and fixed in 10% neutral buffered formalin, embedded in paraffin, sectioned and stained with haematoxylin-eosin. Specific histochemical stainings (PAS, AFOG, PASM) were used for evaluation of microscopic changes in the renal tissue. During necropsy, 4 nematodes were found within the right ventricle of the heart and 2 into the pulmonary artery, and fixed in 70% (v/v) ethanol for later morphological identification (using CIH dichotomous keys [17]) and PCR-based analysis. EDTA blood samples were obtained from the right ventricle of the heart to check for presence of microfilariae by Difil Test technique. A blood sample was also tested by a SNAP-feline *D. immitis *heartworm antigens detecting kit (IDEXX Laboratories inc., USA). Fragments (1 cm length) from different adult worms and microfilaraemic blood samples (0.2 ml each) were molecularly examined. DNA was extracted directly from two worm fragments and two blood samples using the High Pure PCR Template Preparation kit (Roche Diagnostics, Mannheim, Germany). PCR amplification of the 5S ribosomal spacer with primers S2-S16 was performed as described by Favia *et al*. [18]. PCR products were purified using the High Pure PCR Product Purification kit (Roche Diagnostics, Mannheim, Germany) and sequenced on both strands at the BMR-Genomics of Padova using ABPRISM 3700 (Applied Biosystems). The sequencing reactions were analysed using ChromasPro 1.42 and sequences were compared with those available in GenBank using BLAST.

**Figure 1:**
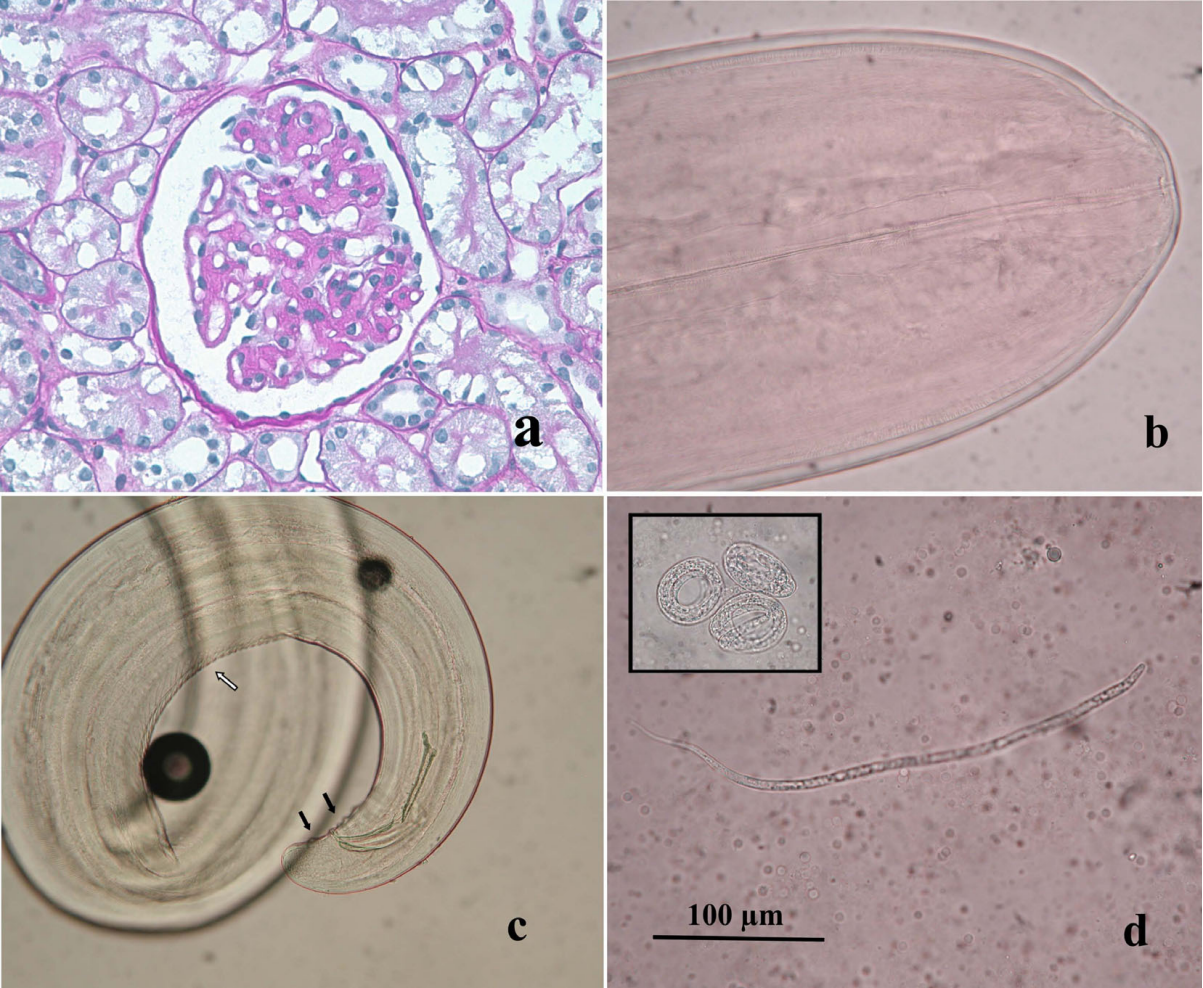
**a) Glomerulus characterized by irregular basement membrane thickening and mild proliferation of mesangial cells and minimal sclerosis (PAS, ×600); b) The small oral orifice of *D. immitis*, with the presence of aring and without lips; c) Rounded posterior extremity of an adultmale of *D. immitis*, with spicules markedly unequal, numerous caudal papillae (black arrows) and crest and striations in the ventral surface (white arrow); d) Microfilaria of *D. immitis *with a pointed anterior extremity and a straight tail**. Many embryos of microfilariae (black square) were observed in the blood collected from the right ventricle of the heart.

Serial renal sections showed different pathological aspects. Glomerular mesangium and glomerular capillary loops were variably thickened by accumulations of an eosinophilic, amorphous to fibrillar material with obliteration of capillary lumina. Glomerular basement membrane presented irregularities in the outer aspect, compatible with spikes positive to PASM stain. Glomerulosclerosis was present. Multifocally, tubules were ectatic and interstitial, often perivascular, aggregates of plasma cells and lymphocytes were present. A diagnosis of chronic multifocal membranoproliferative glomerulonephritis was made (Fig. 1a). Four males and 2 females filarioids were recognised by the tail features. It was possible to measure 2 males and 1 female, while 3 specimens were inadvertently broken during necropsy missing parts of their bodies. The worms had rudimentary buccal capsule with the appearance of a ring and without lips, and lateral alae were absent (Fig. 1b). The female filarioid measured 260 mm in length and 0.85 mm in width (maximum value). Measures of male worms were as follow (first value is that of the shorter worm): total length 160-180 mm, maximum width 0.70-0.75 mm, oesophagus 1.4-1.5 mm. The posterior extremity was rolled in a spiral and caudal papillae were bulky and numerous. Two unequal spicules were present, one 370 × 22 μm and the other 198 × 27 μm in length and maximum width, respectively. The cuticle of filarioids was smooth and lacking in longitudinal ridges, except crest and striations in the ventral surface of the last spiral of the males (Fig. 1c). Worms were identified as *Dirofilaria immitis*. One hundred microfilariae were observed under a light microscope and their features corresponded to that of *D. immitis*. They measured 320-335 μm in length and 5.7-6.7 in width, the cephalic extremity was pointed and the tail straight with a pointed end (Fig. 1d), according to morphological descriptions reported by Manfredi *et al*. [19]. An average value of 2400 microfilariae/ml was calculated by 10 counts serially performed on 10 μl of blood, and several embryos leaked from females specimens were observed (Fig. 1d). Heartworm (*D. immitis*) infection was confirmed by antigen test result and biomolecular analysis. PCR amplifications on adult worms and blood samples showed a single band of 400 bp, therefore excluding the presence of *D. repens*. Sequences obtained both from worm fragments and microfilaraemic blood were identical to each other, and showed a very high degree of homology (99%) with *D. immitis *sequence from domestic dogs [GenBank: M37738] [20].

The leopard arrived at the zoological park in January 2008 from a circus mainly working in the southern Italian regions, where the presence of cHW infection is lower than in endemic areas of the north-eastern Italy [3]. Therefore, over the last 14 months of its life the animal may have been continuously exposed to infected mosquitoes. Although not proved, it is conceivable that *Aedes albopictus*, widely spread in the north-eastern of the peninsula [21,22], may play a non-negligible role in the transmission of *D. immitis *in the area. In this case, it has been demonstrated the capability of *P. pardus pardus *to develop mature heartworms and microfilariae of *D. immitis*. In the only previous report of cHW infection in *P. pardus *[16] a single adult female of *D. immitis *were found in the heart, thus no circulating microfilariae were mentioned. As far as we are concerned, circulating *D. immitis *microfilariae in wild felids were previously detected only in a Clouded leopard (*Neofelis nebulosa*) as results of a routine blood examination performed in the Zoo Negara Kuala Lumpur, Malaysia [9]. Concerning histopathological analysis, immuno-mediated glomerulonephritis is usually observed in animals with high microfilarial counts and long infection periods, due to prolonged release of antigenic material into the blood stream, by inducing in situ formation or trapping of preformed complexes [23]. To our knowledge, in wild felids there is one case report of renal lesions nephropathy correlated with *D. immitis *in a Black-footed cat (*Felis nigripes*) housed at the Central Florida Zoological Park in Sanford, Florida [12]. Although in this case the cHW infection did not appear to be the direct cause of death of the African leopard, there is evidence that only one adult filarioid could cause severe life-threatening disease both in domestic and non-domestic cats [7,24]. It is worthy of note that failures of *intra vitam *diagnoses (*D. immitis *antigens and/or microfilariae detection) could frequently occur because of the long incubation period and the frequent development of few and immature heartworms in felids. This knowledge stresses the importance to provide chemoprophylaxis on wild felids housed outdoors in endemic cHW disease areas, and mainly on males that appear to be at greater risk for cHW exposure than females [7]. Although few experiences are available on this topic, ivermectin preparations for cats are available in the market, and monthly oral administration of 24 μg/kg b.w. has been suggested for the prevention in these animals during the HW transmission season [12]. This latter is about 6 months long in northern Italy (from May to October inclusive). Prevention of cHW disease is undoubtedly preferable to therapy because of the impracticability of the therapeutic protocol in wild felids.

## List of abbreviations

AFOG: Acid Fuchsin Orange G; IUCN: International Union for Conservation of Nature and Natural Resources; PAS: Periodic Acid Schiff; PASM: Periodic Acid-Silver Methenamine.

## Competing interests

The authors declare that they have no competing interests.

## Authors' contributions

SM: carried out necropsy/tissue sampling and drafted the histopathological findings of the manuscript. RC: participated in the parasitological analysis and contributed in drafting the manuscript. LV: helped to draft the manuscript. LA: participated in the histopathological analysis and contributed in drafting the manuscript. AFdR: carried out parasitological analysis and drafted the parasitological findings of the manuscript. All authors read and approved the manuscript.
